# A Legacy to the Metropolitan Asylums Board

**Published:** 1913-03-01

**Authors:** F. A. C. Tyrrell

**Affiliations:** (Assistant Medical Officer Metropolitan Asylums Board Schools for Ophthalmia). 27 New Cavendish Street, Portland Place, W.


Sir,—Adverting to your remarks concerning the legacy recently left to the Metropolitan Asylums Board, I beg to throw out the following suggestion with regard to the application of the money.

A number of children are discharged from the institutions under the Board on account of age. Children who are under treatment for ophthalmia, for instance, are removed by the Guardians, whether cured or not, at the age of sixteen years. These unfortunate young people are consequently compelled to remain in the workhouse infirmaries or to seek situations where they are liable to infect others with a contagious disease. I would suggest that a dispensary such as the Public Health Authority of New York has instituted in connection with the schools should be set up in London for the benefit of these young persons, where they could obtain daily treatment whilst continuing their occupations, thus carrying on the treatment commenced in the institutions under the Board and enabling a cure to be effected. As it is, many of these cases become neglected and eventually end in blindness.—I remain, yours faithfully, F. A. C. Tyrrell, F.R.C.S.

(Assistant Medical Officer Metropolitan Asylums Board Schools for Ophthalmia).

27 New Cavendish Street, Portland Place, W.,
February 24, 1913.

